# Cellular Deubiquitylating Enzyme: A Regulatory Factor of Antiviral Innate Immunity

**DOI:** 10.3389/fmicb.2021.805223

**Published:** 2021-12-13

**Authors:** Sijing Long, Li Yang, Wei Dang, Shuyu Xin, Mingjuan Jiang, Wentao Zhang, Jing Li, Yiwei Wang, Senmiao Zhang, Jianhong Lu

**Affiliations:** ^1^Department of Hematology, National Clinical Research Center for Geriatric Disorders, Xiangya Hospital, Central South University, Changsha, China; ^2^Department of Microbiology, School of Basic Medical Science, Central South University, Changsha, China; ^3^NHC Key Laboratory of Carcinogenesis, The Key Laboratory of Carcinogenesis and Cancer Invasion of the Chinese Ministry of Education, Cancer Research Institute, Central South University, Changsha, China; ^4^China-Africa Research Center of Infectious Diseases, Central South University, Changsha, China

**Keywords:** deubiquitylating enzymes, virus, innate immunity, pattern-recognition receptors, immune evasion, inflammasome, SARS-CoV-2

## Abstract

Deubiquitylating enzymes (DUBs) are proteases that crack the ubiquitin code from ubiquitylated substrates to reverse the fate of substrate proteins. Recently, DUBs have been found to mediate various cellular biological functions, including antiviral innate immune response mediated by pattern-recognition receptors (PRRs) and NLR Family pyrin domain containing 3 (NLRP3) inflammasomes. So far, many DUBs have been identified to exert a distinct function in fine-tuning antiviral innate immunity and are utilized by viruses for immune evasion. Here, the recent advances in the regulation of antiviral responses by DUBs are reviewed. We also discussed the DUBs-mediated interaction between the severe acute respiratory syndrome coronavirus 2 (SARS-CoV-2) and antiviral innate immunity. The understanding of the mechanisms on antiviral innate immunity regulated by DUBs may provide therapeutic opportunities for viral infection.

## Introduction

The innate immune response is a powerful tool for the host cell to sense and resist microbial invasion, such as viruses. Viral proteins and nucleic acids characterize viral pathogen-associated molecular patterns (PAMSs) recognized by pattern-recognition receptors (PRRs) expressed on immune cells. Several major PRRs, including Toll-like receptors (TLRs), cyclic GMP-AMP (cGAMP) synthase (cGAS), RIG-I like receptors (RLRs), and NOD-like receptors (NLRs), as the initiation molecule of immune signal, activating the expression of immune factors such as interferon (IFNs) and proinflammatory cytokines to resist virus infection by inducing specific signaling pathway (Akira et al., 2006; Takeuchi and Akira, 2010). The immune factor can enable cells to establish an effective “antiviral state” and activate adaptive immune responses mediated by T and B cells to collectively resist viral infection (Akira et al., 2006). The effective transmission of immune signals is assisted by multiple regulatory actions, including the ubiquitin system, a dynamic network composed of ubiquitin enzymes, deubiquitylating enzymes, ubiquitin molecules, and modified substrate proteins (Mevissen and Komander, 2017).

Ubiquitin is a small protein of 76 amino acids that exist in eukaryotes, it modifies the target protein under the catalysis of a series of enzymes, including ubiquitin-activating enzymes (E1), ubiquitin-conjugating enzymes (E2), and ubiquitin ligases (E3). With the specific treatment of the E3 enzyme, the ubiquitin molecules can bind to a target protein in a manner of individuals or the form of the chain. Such modification is called mono-ubiquitination modification or poly-ubiquitination modification (Liu et al., 2005). The most common type of polyubiquitination is the binding of the lysine site in the middle of the ubiquitin protein (including K6, K11, K27, K29, K33, K48, and K63), and the other type is the end-to-end binding of the ubiquitin molecules, support linear ubiquitination. The most widely studied is the polyubiquitin chain mediated by K48- and K63-linked polyubiquitin chains, the K48-linked ubiquitin chains mainly guide proteins to 26S proteasome for degradation, while the K63-linked ubiquitin chains have non-degradation functions, involving activation of signal factors, the interaction between proteins, DNA damage response (Hershko and Ciechanover, 1998). Same to other post-transcription, the ubiquitin process is reversible. The deubiquitinating enzymes (DUBs) can antagonize the effect of ubiquitin molecular by specifically identifying and removing conjugated ubiquitin from the target protein, and there are nearly 100 kinds of deubiquitinating enzyme genes in the human genome, based on the unique structure, DUBs can be classified into five members: the ubiquitin-specific proteases (USPs), the ovarian tumor-related proteases (OTUs), the ubiquitin carboxy-terminal hydrolases (UCHs), the Machado–Joseph disease protein domain proteases (MJDs), and the Jad1/Pad/MPN-domain-containing metalloenzymes (JAMMs), which are involved in the regulation of cellular biological functions (Komander et al., 2009; Clague et al., 2019). Accumulating evidences has gradually revealed the role of DUBs in innate immunity, which regulates the PRRs mediated antiviral responses and the transmission of signal pathways (Zong et al., 2021). It also modulates the activation of the NLR Family pyrin domain containing 3 (NLRP3) inflammasome. NLRP3, a member of the cytoplasmic PRRs receptor NLR family, could be activated by PAMPs and danger-associated molecular patterns (DAMPs) to promote antiviral responses mainly by releasing inflammatory factors leading to pyroptotic cell death (Broz and Dixit, 2016). However, this regulation is not unique to the host, and viruses can hijack the cellular DUBs to regulate immune signals for their benefits, like achieving immune evasion, facilitating self-replication. This review discusses the DUBs participating in the regulation of antiviral immunity signaling and how viruses manipulate the immune regulation of cellular DUB to promote their interests.

## Deubiquitination in Ikk Activation

Activation of the inhibitor of κB kinases (IKK) is critical for PRR signaling. A class of IKK complexes, composed of two catalytic subunits IKKα and IKKβ, a regulatory subunit: NF-κB essential modulator (NEMO, also known as IKK γ). Upon activation, IKK phosphorylates IKBα, causing IKBα degraded by the proteasome pathway, NF-κB can be reactivated and transferred into the nucleus, facilitating the induction of proinflammatory cytokines. While IKKε and TANK-binding kinase 1 (TBK1), phosphorylates IRF7 and IRF3, respectively, promotes that they enter the nucleus as dimers and initiate the expression of type I IFNs (Chen, 2012; Jin et al., 2014).

TBK1 is an important signaling adaptor in type I IFN signaling, and its activation needs to form complexes with mitochondrial antiviral-signaling protein (MAVS) or stimulator of interferon genes (STING), and TNF receptor-associated factor (TRAFs) for its activation (Fang et al., 2017). Moreover, the ubiquitination modification is also required (Zeng et al., 2009). A variety of viruses can attenuate interferon production by inhibiting TBK1 activation. The membrane-anchored papain-like protease (PLpro) domain (PLpro-TM) of SARS-CoV interacts with the STING-TRAF3-TBK1 complex and disrupts the interaction between the components in the complex. In addition, SARS PLpro-TM decreases the levels of ubiquitination of TBK1 in the STING-TRAF3-TBK1 complex (Chen et al., 2014). The nonstructural proteins 5 (NS5) of Zika virus (ZIKV) combines with the ubiquitin-like domain of TBK1 and inhibits the complex of TBK1 and TRAF6, and subsequently diminishes the phosphorylation, nuclear translocation, and activation of IRF3 subsequently (Lin et al., 2019). Some viruses may inhibit TBK1 activation at the ubiquitination level. For example, the membrane (M) protein of severe acute respiratory syndrome coronavirus 2 (SARS-CoV-2) interacts with and catalytic the K48-linked ubiquitination of TBK1, promoting its degradation (Sui et al., 2021). The leader proteinase [L(pro)] of foot-and-mouth disease virus (FMDV) and Seneca Valley Virus (SVV) 3C protease (3Cpro) are viral proteins with DUB activity, both of which can significantly inhibit the ubiquitination of TBK1 and reduce the expression of interferon-related genes (Wang et al., 2011; Xue et al., 2018). Therefore, the regulation of TBK1 by DUB is somewhat necessary, for fine-tuning the transmission of antiviral signals.

E3 ligase DTX4, TRIP, and TRIM27 are responsible for inducing the K48-linked ubiquitin chains and degradation of TBK1 (Zhang et al., 2012). Several DUBs have been demonstrated involved in modulating the K48-linked ubiquitin chains of TBK1. The complex of USP1 and UAF1 (USP1-associated factor 1) removes the K48-linked ubiquitin chain after binding to TBK1. USP1-UAF1 complex increased TLR3/4 and RIG-I mediated IFN-β production, and the discovered USP1-UAF1 inhibitor ML323 can reduce interferon expression and promote viral replication both *in vivo* and *in vitro* (Yu et al., 2017). USP7 was found to bind to TRIM27 and this binding effect was significantly enhanced after viral infection. USP7 stabilizes TRIM27 by removing the K48-linked ubiquitin chain of TRIM27 and increases the degradation effect of TRIM27 on TBK1 (Cai et al., 2018). USP38 has a special effect on the K48-linked ubiquitin chains of TBK1. It cleaves the K33-linked ubiquitin chain at lys670 of TBK1 and allows the E3 enzyme DTX4, TRIP to catalyze the formation of the K48-linked ubiquitin chains at the same position in an NLRP4-dependent manner. USP38 knockdown has enhanced the ubiquitination of K33-linkage poly-ubiquitin chains, but the formation of the k48-linked ubiquitin chain and the degradation of TBK1 are disrupted, indicating that the effects of DTX4 and TRIP on TBK1 depend on the presence of USP38. Collectively, USP38 could promotes TBK1 proteasome degradation and maintains the homeostasis of the antiviral response (Lin et al., 2016a).

K63 polyubiquitination contributes to TBK1 activation (Li et al., 2011). Three DUBs were shown toward the K63-linked ubiquitin chains of TBK1. The ubiquitin-editing enzyme A20 and its adaptor protein TAX1BP1 antagonistic K63-linked polyubiquitination of TBK1 to block its activity, inhibiting the transmission of antiviral signals (Parvatiyar et al., 2010). Another study showed that USP2b can also deconjugate the K63-linked ubiquitin chain of TBK1, overexpression of USP2b contributes to the enhanced replication of vesicular stomatitis virus (VSV; Zhang et al., 2014). In addition, USP15 interacts with TBK1 and inhibits its activity only in the presence of ubiquitin-conjugating enzyme UBE2C. UBE2S possesses the E3 ligase activity, it regulates the innate immune responses by targeting the K63-linked ubiquitin chain at K30/k401 of TBK1, regardless of its E2 or E3 enzyme activity, but by recruiting USP15 to form the UBE2C-USP15-TBK1 complex. This complex composed of USP15 increases virus replication during infection (Huang et al., 2020). In total, these results indicated that DUBs play an indispensable role in TBK1 activation.

Atypical, K6-linked ubiquitination is important for the DNA binding ability of IRF3 and the induction of target genes (Ndoja et al., 2014; Li et al., 2019b), and it can be cleaved by OTUD1 after viral infection. Moreover, the ovarian tumor domain allows the OTUD1 to separate IRF3 from the promoter region of the target gene and inhibits the normal production of interferon, while it does not affect the normal dimerization of IRF3 and translocate into the nucleus. After viral infection, Otud1^−/−^ mice produced higher IFN-I and proinflammatory cytokines and were more resistant to the infection of herpes simplex virus (HSV) and VSV (Zhang et al., 2020). In brief, this article revealed that DUBs are capable of regulating the noncanonical ubiquitination to affect IRF3 regulation of target genes and interferon production.

OTUD6B, another member of the OTUD family, was found to inhibit TRAF6-induced K63-linked polyubiquitination of IRF3 and IRF7 in zebrafish, depending on the role of the OTU domain. Besides, OTUD6B can also inhibit the binding of TBK1 to IRF3 and IRF7 and damage the phosphorylation of IRFs molecules. Mutated OTUD6B can improve the antiviral infection ability and survival rate of zebrafish in spring (Zhou et al., 2021). USP22 is a kind of DUB capable of shuttling between cytoplasm and nucleus, and it promotes the entry of IRF3 into the nucleus by regulating the ubiquitination and stability of intermediate protein KPNA2. KPNA2 acts as a transporter to help IRF3 undergo nuclear heterotopic. USP22 inhibits the degradation of KPNA2 through NDP53 mediated selective autophagy, which stabilizes the nuclear translocation of IRF3 (Cai et al., 2020). These findings may promote the research progress of the mechanism of DUBs regulating the nuclear ectopic mechanism of IRF3.

## The Multiple Roles of Dubs in Prrs Mediated Antiviral Signaling

Deubiquitylating enzymes precisely control the transmission of PRR signaling. Some opposite regulatory results of DUB reflect the bidirectional regulation of DUB on PRR signaling. Special protein interaction domains are one of the reasons that DUBs negatively regulate PRR signaling. A characteristic of USP18 is that it has a domain interacting with IFNs receptor IFNAR2. USP18 blocks IFNAR2 binding with its original ligand JAK1, hampering the production of downstream interferon-stimulated genes (Malakhova et al., 2006). In addition, USP18 could deconjugate K63-linked ubiquitin chains of both TAK1 and NEMO to inhibit IFNs production mediated by the NF-κB pathway (Liu et al., 2013; Yang et al., 2015). In the aspect of promoting immune signaling, USP18 mostly acts as a molecular scaffold, and it serves as a scaffold protein to recruit USP20 to bind and remove the K48-linked ubiquitin chain of STING or induce the relocation of TRIM31 from the cytoplasm to mitochondria and promoting the interaction between TRIM31 and MAVS, facilitating the formation of more MAVS aggregates (Zhang et al., 2016; Hou et al., 2021).

Preference for different catalyzed substrates and specific ubiquitin branches ultimately contribute to the bidirectional regulation of DUBs in antiviral immune signaling, such as USP15. In RLR-mediated immune signaling, E3 ligase TRIM25 catalytic K63-linked ubiquitylation of RIG-I, promoting it undergoes oligomerization and interacts with MAVS efficiently and stimulate downstream signaling (Gack et al., 2007). Multiple sites of the SPRY domain in TRIM25 experience K48-linked polyubiquitylation that can be mediated by E3 ligase complex LUBAC (Inn et al., 2011). Studies have found that ectopic expression of USP15 effectively reduces LUBAC-mediated Lys48-linked polyubiquitin chains of TRIM25, leading to its stabilization. The stabilizing effect of USP15 on TRIM25 indirectly upregulated the expression of RIG-I in infected cells. In responsible for virus infection, the expression of USP15 is required for TRIM25 and RIG-I induced IFNs production (Pauli et al., 2014). However, the regulation of USP15 in IFNs signaling seems not constant. A follow-up study showed that USP15 exhibits negative regulation in innate immunity. In this study, overexpression USP15 dampened the transcription of IFN-β, which directly removed the K63-linked ubiquitin chains of RIG-I, and the catalytic activity of USP15 is dependent on histidine 862, critical catalytic properties of the USPs. In addition, mutation of the catalytic residue does not abolish USP15 on IFN signaling antagonism completely. It is indicated that still have some USP15 inhibits IFNs production independent of its DUB activity. This part of USP15 is associated with RIG-I in a manner of physical interaction, which hindered the interaction between RIG-I and IPS-1 (an adaptor triggering RIG-I mediated IFNs signaling by binding with the N-terminal CARDs of RIG-I) (Zhang et al., 2015). Furthermore, USP15 can be recruited by ubiquitin-conjugating enzyme UBE2S (E2) to deubiquitylates TBK1, inhibiting type I IFN production (Huang et al., 2020). These two contradictory results reflect the tight regulation of DUBs in antiviral signals, although dealing with RNA virus infection in both cases, it probably worked at different periods.

Another example is cylindromatosis (CYLD). CYLD was originally considered a tumor suppressor gene mutated in familial cylindromas (Bignell et al., 2000). It has been extensively studied in the antiviral immune response, negatively regulating most PRR signaling. The C-terminal part of CYLD contains a class of catalytic domains homologs to the USP family, which mediated the affinity of CYLD for the K63-linked ubiquitin chain and linear ubiquitin chain (Komander et al., 2008; Sato et al., 2015). It inhibited the NF-κB pathway by removing the K63-linked ubiquitin chain on TRAF2, TRAF6, and the linear ubiquitin chain of NEMO (Kovalenko et al., 2003). Recently, the E3 ligase HUWE1 has been found to protect the TRAF6 from the deubiquitylation of CYLD by generating k48 branched on the K63-linked polyubiquitin chain of TRAF6 (Ohtake et al., 2016). In addition, a variety of PRRs signaling factors including RIG-I, TBK1, STING, TRAF7, NEMO, and RIP1 are regulated by CYLD. CYLD toward the K63-linked ubiquitin chain of RIG-I and utilized by Optineurin (Optn) to inhibit the enzyme activity of downstream kinase TBK1, which efficiently dampens the basic signal of IFNs induced by RIG-I (Friedman et al., 2008; Zhang et al., 2008). In a yeast two-hybrid screen, identifying a glycoprotein existing on the cell membrane, SDC4, interacts with RIG-I and CYLD through its carboxyl-terminal intracellular domain, promoting the deubiquitylation of RIG-I by CYLD and facilitating viral replication. What is more, the auxiliary effect of SDC4 on CYLD may also affect the membrane redistribution of RIG-I in a perinuclear pattern under viral infection (Lin et al., 2016b). In most cases, CYLD plays a stabilizing role in immune signals and prevents excessive activation. In particular, researchers have recently found that CYLD acts as a specific checkpoint of the STING-mediated antiviral signaling after DNA virus infection. CYLD transfers to Golgi from the endoplasmic reticulum (ER) to remove the K48-linked ubiquitin chain of STING, enhancing the innate antiviral response (Zhang et al., 2018).

Nowadays, many DUBs have been identified to regulate PRR signaling bidirectionally through similar mechanisms. It is sufficient to prove that DUBs does not play a single role in antiviral signals; the immunomodulatory effects of DUBs are diverse and complex, which cannot simply be defined as immune activators or suppressors. It also shows that the regulation process of antiviral signal needs to be controlled accurately. These DUBs that play a dual role in PRRs signaling are summarized in Table 1.

**Table 1:** The multiple roles of deubiquitylating enzymes (DUBs) in pattern-recognition receptor (PRR) signaling.

DUBs	Substrate	Positive regulation	Negative regulation	References
USP13	STING, STAT1	Stabilizing STAT1 expression	Removing K27/33-linked polyubiquitin chains from STING	Yeh et al., 2013; Sun et al., 2017
USP14	RIG-I, CGAS	Recruited by TRIM14 to stabilize CGAS and promote IFNs production.	Removing K63-linked polyubiquitin chains from RIG-I	Chen et al., 2016; Li et al., 2019a
USP15	RIG-I, TBK1	Stabilizing the expression of TRIM25	Removing K63-linked polyubiquitin chains from RIG-I; Recruited by UBE2S to deubiquitinating TBK1	Pauli et al., 2014; Zhang et al., 2015
USP18	TAK1, NEMO, STING, MAVS, IFNAR2	Acting as a Scaffold protein to recruit USP20 and TRIM31, stabilizing STING and MAVS, respectively.	Interrupting the interaction between IFNAR2 and JAK1; removing K63-linked polyubiquitin chains from TAK1 and NEMO	Malakhova et al., 2006; Liu et al., 2013; Yang et al., 2015; Zhang et al., 2016; Hou et al., 2021
USP27X	RIG-I, CGAS	Removing K48-linked polyubiquitin chains from CGAS	Removing K63-linked polyubiquitin chains from RIG-I	Guo et al., 2019; Tao et al., 2020
OTUD4	Myd88, TRAF6, MAVS	Removing K48-linked polyubiquitin chains from MAVS	Deubiquitinating K63-linked polyubiquitin chain from TRAF6 and Myd88	Zhao et al., 2018; Liuyu et al., 2019; Liu et al., 2020
OTUD5 (DUBA)	TRAF3, STING	Removing K48-linked polyubiquitin chain on STING	Removing the K63-linked polyubiquitin chain on TRAF3	Kayagaki et al., 2007; Guo et al., 2021b
OTUB1	RIG-I, TRAF3, TRAF6	Deubiquitinating K48-linked ubiquitin chain or forming a complex with UBCH5c to activate RIG-I	Removing K63-linked polyubiquitin chains on TRAF3/TARF6.	Li et al., 2010; Jahan et al., 2020
CYLD	TRAF2, TRAF6, NEMO, RIP1, TBK1, STING, RIG-I	Removing K63-linked polyubiquitin chains on RIG-I and TRAF2/6; Removing linear ubiquitin chain on NEMO; Inhibiting the enzymatic activity of TBK1	Removing K48-linked polyubiquitin chains on STING.	Yoshida et al., 2005; Zhang et al., 2006; Friedman et al., 2008; Genin et al., 2015; Lork et al., 2018; Zhang et al., 2018

## Posttranslational Modification Affected the Regulation of Dubs on Prr Signaling

Deubiquitylation is a sophisticated event, resulting from the catalytic activity and function of DUBs are also modulated by post-translational modifications except for the functional diversity of DUB (Mevissen and Komander, 2017). This modification may influence DUBs to regulate the antiviral immune response.

Prior work discovered that rapid and transient phosphorylation of CYLD is necessary for the ubiquitination of TRAF2 and activation of JNK (Lork et al., 2018). It requires co-expression with IKKγ. Phosphorylation of Ser418 of CYLD inhibits CYLD-mediated TRAF2 deubiquitination, promoting TNF-induced gene expression (Reiley et al., 2005; Hutti et al., 2009). Recent evidence demonstrates a new phosphorylation site, Ser568 in CYLD, which could stimulate CYLD toward K63-linked polyubiquitin chains. Ser568 works with Ser418 to facilitate CYLD-dependent regulation of TNFR1 and NOD2 signaling. The author also identified that CAP-Glys3 regions outside of the catalytic domain in CYLD regulate their activity and are responsible for CYLD-mediated NOD2 signaling in cells. These findings indicated that phosphorylation of the residues in CYLD is critical for innate immune response. TNF-RSC and NOD2-SC is a transient multi-protein complex formed stimulated by TNF and NOD2 in the cytoplasm upon cellular stimulation. CYLD was recruited to the two complexes together with A20, limiting gene activation cooperatively. The difference is that they have opposite effects on the stability of the M1 chain in the complex. CYLD unravels M1 chains, increasing the sensitivity of cells to TNF-induced cell death, whereas A20 prevents the removal effect, thus inhibiting cell death (Elliott et al., 2021).

On the other hand, phosphorylation can increase the regulatory effect of A20 on antiviral immunity. Research shows that IKKβ phosphorylated A20 at Ser381 both *in vitro* and *in vivo*, which enhances the ability of A20 in inhibiting the NF-κB pathway (Hutti et al., 2007).

## The Role of Dubs in Nlrp3 Inflammasome Mediated Antiviral Signaling

**Figure 1:**
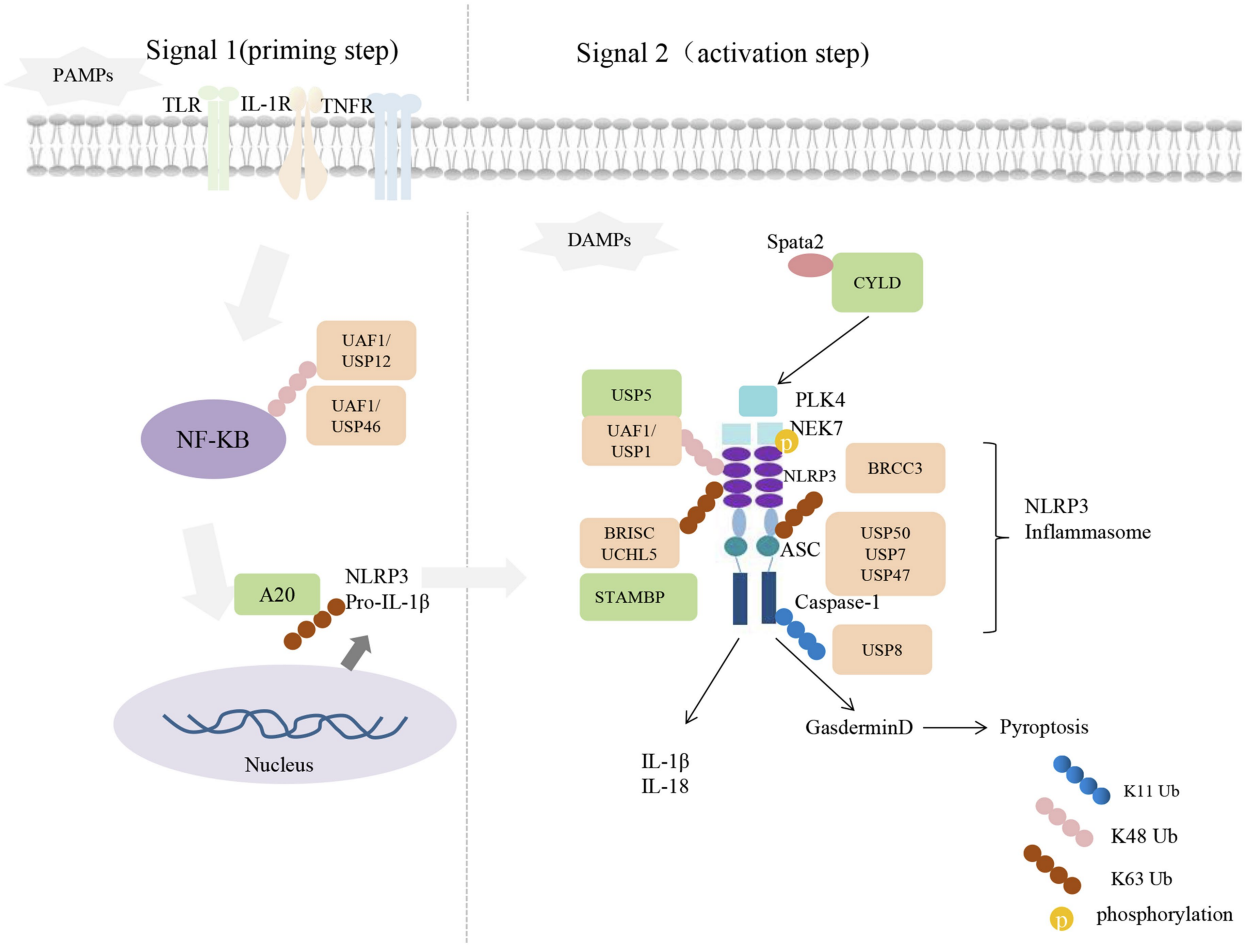
Deubiquitylating enzymes in the activation of NLR Family pyrin domain containing 3 (NLRP3) inflammasome. The expression of NLRP3 and pro-IL-1β induced by NF-κB as the priming signal of NLRP3 inflammasome activation. Signal 2: with the stimulate of danger-associated molecular patterns (DAMPs), NEK7 binds with NLRP3, promoting its oligomerization and activation completely. Activated NLRP3 interacts with apoptosis-associated speck-like protein (ASC) through the N-terminal Pyrin domain to recruit pro-caspase-1, enhancing autoproteolytic activation of caspase-1. Cellular DUBs that positive or negative regulate the signaling are shown with orange or green color, respectively.

NLRP3 and absent in melanoma 2 (AIM2) recruit apoptosis-associated speck-like protein (ASC) and caspase-1, forming NLRP3 inflammasome. NLRP3 inflammasome-mediated antiviral responses could through the maturation and release of interleukin (IL)-1 and IL-18 to induce cell death (Man and Kanneganti, 2016). The activation of NLRP3 inflammasome is induced in response to RNA viruses such as SARS-CoV and IAV (Tate and Mansell, 2018; Siu et al., 2019). Mice lacking NLRP3 or other NLRP3 inflammasome complex components were shown to be susceptible to IAV infection (Tate and Mansell, 2018). Different DUBs play specific regulatory roles in NLRP3 inflammasome activation, strictly controlling inflammasome assembly and activation. Here, we used Figure 1 to summarize the regulatory role of DUBs on the NLRP3 inflammasome.

### Positive Regulators of DUBs on NLRP3 Inflammasome

BRCC3, BRCC3 complex components ABRO1, USP50, USP7, USP47, and UAF1 are DUBs that contribute to the activation of NLRP3 inflammasome. Treated mouse macrophages with the DUB inhibitor G5 showed the secretion of IL-1β was decreased, more importantly, drove NLRP3 ubiquitination, and the activation of caspase-1 was inhibited, indicating that deubiquitylation may affect NLRP3 inflammasome activation. By screening the expression library of DUB determined that BRCC3 (named BRCC36 in humans) is the critical DUB affecting NLRP3 activation; it specifically binds to the LRR domain of NLRP3 to mediate deubiquitylation and inhibit NLRP3 degradation, which may act on the K63-linked polyubiquitin chain of NLRP3. In comparison, it is not clear about the *in vivo* effect of BRCC3 on the deubiquitylation of NLRP3 (Py et al., 2013).

ABRO1, a component of the BRCC3 complex (also known as BRISC), was found to stabilize BRCC3 and is necessary for NLRP3 complex formation, NLRP3 inflammasome subunits ASC oligomerization, and downstream caspase-1 activation. ABRO1 binds to NLRP3 in a phosphorylation-dependent manner, which facilitates the deubiquitylation of BRISC to NLRP3. Importantly, the ABRO1-knockout mice exhibit the same phenotype as mice lacking the active ingredient of BRISC (Ren et al., 2019), indicating the importance of ABRO1 on the catalytic activity of BRCC3 complex and the activation of inflammasome. Chemical inhibition of USP7 and USP47 can block spot formation and oligomerization of ASC and affect the total ubiquitination level of NLRP3, but the K48- and K63-linked ubiquitin chains on the NLRP3 after inflammasome activation are not affected. Although USP7 affects the NF-κB pathway, it does not initiate TLR4-induced inflammasome activation in this way. Furthermore, activated inflammasomes also regulate USP7 and USP47 activity, which forms a positive feedback pathway for resistance to pathogen infection (Palazon-Riquelme et al., 2018).

UAF1 is involved in NLRP3 inflammasome activation by combining with USP12 or USP46 to form a deubiquitinase complexes. In the initial step of inflammasome activation, the complex of UAF1/USP12 and UAF1/USP46 enhances NF-κB signal transduction by removing the K48-linked ubiquitination of p65, which enhances NF-κB-mediated transcription of NLRP3 and cytokines. On the other hand, UAF1 also binds USP1 to selectively remove the K48-linked polyubiquitylation of NLRP3, contributing to subsequent assembly and activation of NLRP3 inflammasome (Song et al., 2020).

Apoptosis-associated speck-like protein spot formation and oligomerization require the participation of USP50; it cleaves the K63-linked ubiquitin chains of ASC, which may prevent the degradation of NLRP3 inflammasome through autophagy. In addition, depletion of USP50 in both human THP-1 and mouse macrophages affected spot formation and oligomerization of ASC decreases NLRP3 activity, which resulted in reduced secretion of IL-1β and IL-18 (Lee et al., 2017).

### Negative Regulators of DUBs on NLRP3 Inflammasome

Several DUBs, including A20, Spata2-CYLD, and STAMBP, inhibit NLRP3 inflammasome activation. A20 (also known as TNFAIP3) is an NF-κB-responsive gene and is also required for terminating PRR-mediated innate immunity (Boone et al., 2004; Coornaert et al., 2009). It has been reported that A20 blocks NLRP3 inflammasome activation by inhibiting the ubiquitination of pro-Interleukin-1β protein complexes (Duong et al., 2015). In addition, the inhibition of A20 on NLRP3 inflammasome may prevent the development of several inflammatory diseases. For example, negative regulation of NLRP3 inflammasome by blocking caspase-1 activation, may preventing further deterioration of rheumatoid arthritis (Walle et al., 2014). Another study revealed that haploidy deficiency of A20 (HA20) caused by TNFAIP3 deletion mutation induces inflammatory disease (Aeschlimann et al., 2018). P. (Lys91*) mutation is a new member of HA20 mutations that impair the interaction between A20 and proteins, resulting in loss of A20 inhibition of NF-κB and NLRP3 inflammasome. Enhanced caspase-8-dependent NLRP3 inflammasome reactivity leads to the secretion of IL-1 β and IL-18 in HA20 patients (Rajamaki et al., 2018).

In addition, the activation of NLRP3 inflammasome is inhibited by the centrosome protein Spata2, a well-known CYLD companion protein, which can recruit CYLD to deubiquitinate centrosome-spindle kinase PLK4 in the centrosome. It contributes to the binding of PLK4 to NEK7 and phosphorylation of NEK7 at Ser204, weakening the binding of NEK7 to NLRP3, which is critical for the activation of NLRP3 inflammasome. Both PLK4 knockdown and Ser204 mutation of NEK7 enhanced NLRP3 activity (Yang et al., 2020).

The deubiquitinase STAMBP is also a negative regulator of NLRP3. Knockout of STAMBP in monocytes increases LPS or TLR-induced chemokine expression. The difference is that STAMBP does not affect the protein abundance of NLRP3, but STAMBP deficiency enhances the K63-linked ubiquitin chain of NLRP3, and the non-degraded STAMBP ubiquitination process favors NLRP3 inflammasome activation (Bednash et al., 2021).

## Dubs Regulate the Antiviral Immune Signaling in the Autophagy-Lysosomal System

In addition to reversing the ubiquitin signal added to the substrate protein, DUBs also regulate substrates through autophagy pathways. Screening the potential NLRP3 partner in co-immunoprecipitation (Co-IP) experiments have identified USP5 as a DUB that interacts with NLRP3 and locates in the lysosome. USP5 functions as a scaffold to recruit the E3 enzyme MARCHF7/MARCH7, promoting the K48-linked ubiquitination of NLRP3 and subsequent autophagy-lysosomal degradation. Increased USP5 expression in mice reduced IL-1β and PMN infiltration in aluminum-induced peritonitis (Cai et al., 2021). Data from another study have found that USP19 regulates kinase TBK1 lysosomal degradation; it did not change the ubiquitination of TBK1, and mutation with enzyme activity of USP19 still can bind with TBK1 and promote its degradation. In comparing with TBK1, amino acid sequences in different species have found a conserved CMA-motif (KFDKQ) dependent on key protein HSPA8 and LAMP2A, which is associated with the selective selection protein degradation process. USP19 interact with TBK1, HSPA8 as well as LAMP2A, promoting the expression of HSPA8 and LAMP2A. Knockdown of HSPA8 or LAMP2A disrupts CMA could restore the TBK1 protein level in the case of USP19 expression (Zhao et al., 2021). Moreover, USP15 endows a role in the negative regulation of antiviral immune signaling mediated by TNF α and IL-1β *via* autophagy pathway, in which USP15 targets the subunit of TAK1 complex, TAB2, and TAB3. The interaction enhanced between USP15 and TAK1 complex upon stimulated by TNFα. USP15 induce unraveling of lysine 48-linked TAB2 ubiquitination or prevents lysosome-related TAB1 from degradation in a deubiquitination-dependent manner. On the other hand, it promotes NBR1-mediated selective autophagic degradation of TAB3, independent of its deubiquitinase activity. The differential regulation consistently maintained the TAK1-TAB complex and potentiated NF-κB activation induced by TNF-α and IL-1β (Zhou et al., 2020). In addition, USP50 can also inhibit NLRP3 inflammasome degradation through the autophagy pathway as mentioned above (Lee et al., 2017). The results indicated that other protein’s degradation process is also regarded as an effective way for DUBs to regulate the stability of substrate protein, which affects the transmission of host antiviral signaling.

## Cellular Dubs Exploited By Viruses To Support Self-Replication and Immune Evasion

**Figure 2:**
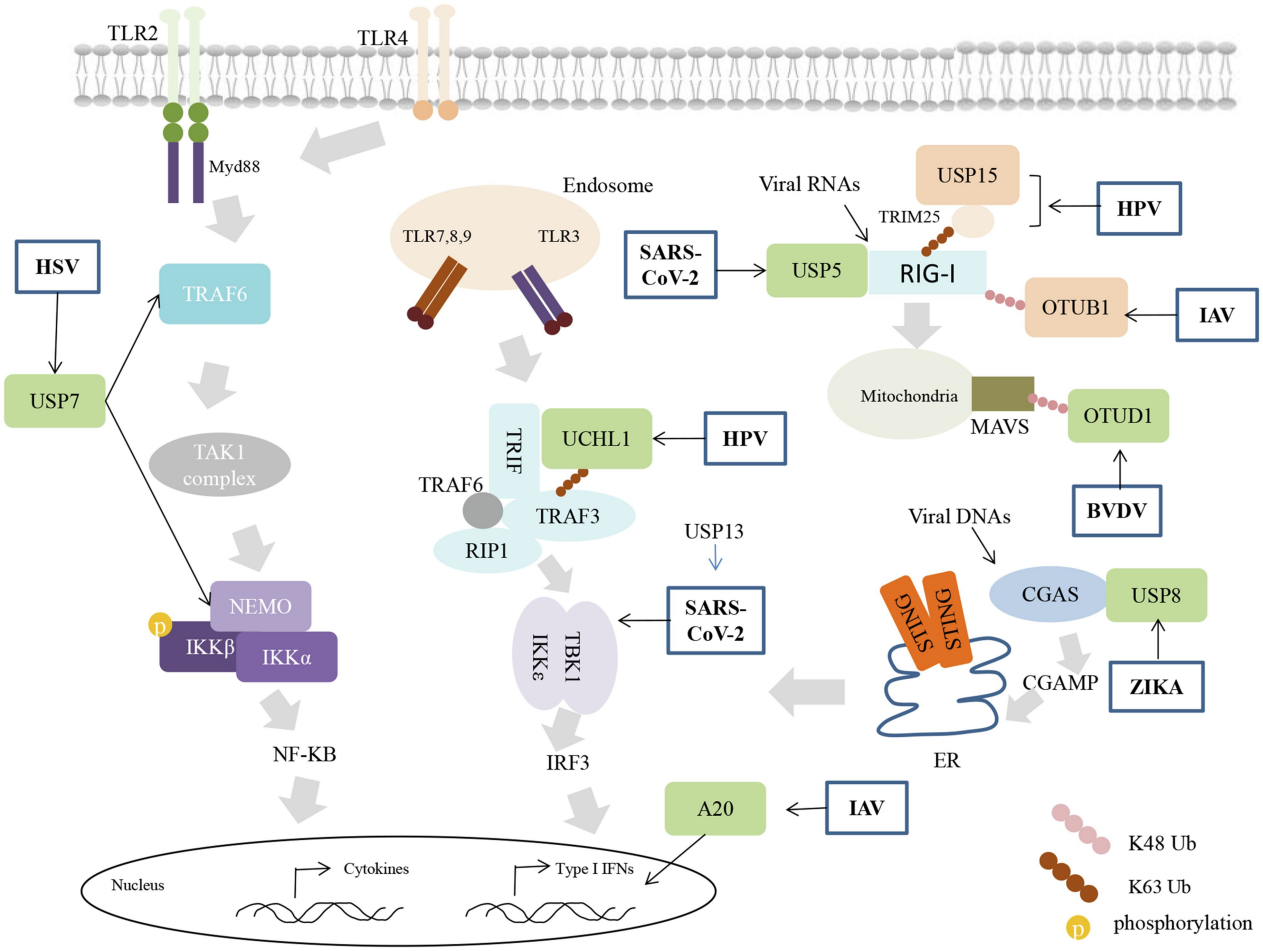
Viruses inhibit innate immunity using cellular DUBs activity. Viral infection induces a cascade of immune signals resulting in the expression of interferon (IFN)-I. Viruses utilize the host’s DUB to block different steps of the antiviral response. Cellular DUBs that positive or negative regulate the signaling are shown with orange or green color, respectively.

The innate immune response is a dynamic process of interaction between host and virus. Along the way, viruses have evolved strategies to prepare for immune evasion and their replication. Although viruses can also encode proteins with DUB activity (Kumari and Kumar, 2018), they often utilize cellular DUB for benefits, as illustrated in Figure 2.

Herpes simplex virus utilizes USP7 to maintain its latent state. HSV immediate-early protein (ICP0) blocks TLR-dependent inflammatory responses mainly through USP7. ICP0 mediates the transfer of USP7 from the nucleus to the cytoplasm, where USP7 binds and deubiquitinates TRAF6 and NEMO, weakening the host’s innate immune response to HSV-I infection (Daubeuf et al., 2009). In addition, ICP0 induces the K48-linked ubiquitination of BRCC36 to indirectly lead to downregulation of the IFN receptor IFNAR1, blocking IFN at its functional stage (Zhang et al., 2021b).

Epstein-Barr virus (EBV) can exist in most people in a latent state, inseparable from its ability to escape and destroy the innate immune system (Xin et al., 2019; Dang et al., 2021). EBV oncoprotein LMP1 promotes the expression of IRF7, which has been implicated in IFNs signaling in previous studies (Ning et al., 2005). Interestingly, A20 induced by LMP1 has a negative regulatory effect on IRF7 by directly binding to reduce ubiquitination of IRF7 and inhibit its activity (Laherty et al., 1992; Ning and Pagano, 2010), which seems that IRF7 is subject to a variety of complex regulation in the case of EBV infection and it may benefit the latent infection of EBV in the host and promote the carcinogenic effect on the host.

The E6 protein is one of the high-risk human papillomaviruses (hrHPV) proteins with oncogenic properties. In the RLR-mediated signaling pathway, E6 forms a three-molecule complex with TRIM25 and USP15, which triggers the K48-linked ubiquitination and proteasomal degradation of TRIM25, attenuates the TRIM25-mediated K63-linked ubiquitination of RIG-I. USP15 is known to cut the K48-linked ubiquitin chain of TRIM25 to stabilize its expression, while E6 intervention may compete with USP15 to regulate TRIM25, impeding the original RIG-I signal activation (Chiang et al., 2018). UCHL1 a thiol protease could bind with monoubiquitin and prevent its degradation from the lysosome pathway, except for hydrolyzing K48-linked ubiquitylation, UCHL1 could elaborate K63-linked polyubiquitin chains on substrate molecular (Leroy et al., 1998; Bilguvar et al., 2013). The regulatory function of UCHL1 in antiviral signaling was discovered based on the study of immune evasion of HPV. The researcher utilizes a unique mode of hrHPV (high-risk HPV) infection, including the PRR signaling in non, newly, persistently hrHPV-infected keratinocytes, and found that PRR induced production of pro-inflammatory cytokines, IFNs, chemokines are suppressed in the case of active infection with hrHPV. Moreover, it depends on the attenuation of antiviral signaling by cellular UCHL1. UCHL1 inhibited the K63-linked ubiquitination of TRAF3 and reduced the number of TRAF3-TBK1 complexes. Even more, it promotes NEMO degradation and inhibits the phosphorylation of P65 and IRF3, effectively blocking the production of inflammatory factors and interferons (Karim et al., 2013). How HPV utilizes UCHL1 to inhibit the immune response is not clear yet, but the presence of UCHL1 greatly reduces the clearance of HPV infected cells by the immune system.

The NS1 protein of Influenza A Virus (IAV) regulates diverse functions, including but not limited to virus mutation, replication, and transmission (Krug, 2015). In A549 cells, NS1 dramatically induces the expression of A20, a typical negative regulator of immune signals. Highly expressed A20 suppressed IAV-induced the expression of IRF3 and type I IFNs, and lung epithelial cells and myeloid cells lacking A20 can resist IAV infection (Feng et al., 2017). The results indicated that induced A20 is related to viral virulence and inhibits the innate immune responses after IAV infection, consistent with previous reports that A20 deficient macrophages are hyperresponsive to IAV infection, concomitant with higher production of proinflammatory cytokines and type I interferon (IFN-I). *In vivo*, A20^−/−^ mice show increased alveolar macrophages and neutrophils (Maelfait et al., 2012). In addition, IAV can block OTUB1 induced immune responses. OTUB1 removes the K48-linked ubiquitin chain of RIG-I through enzymatic activity. In addition, it can form a complex with the E2 enzyme UBCH5c to inhibit the K48-linked ubiquitination of RIG-I. This dual mechanism of OTUB1 greatly promotes the activation of immune signals. The NS1 proteins trigger OTUB1 degradation through the proteasome pathway, inhibiting the activation of RIG-I signals during IAV infection (Jahan et al., 2020).

Infection of ZIKV triggers the activation of NLRP3 inflammasome (Wang et al., 2018). The NS1 protein of the virus recruit of USP8 to remove the K11-linked polyubiquitin chains at lys134 of caspase-1, the enhanced stabilization of caspase-1 further promotes cleavage CGAS, which inhibits type I IFN signaling and benefits ZIKA infection (Zheng et al., 2018).

Bovine viral diarrhea virus (BVDV) infection induced the expression of DNA damage-inducible transcript 3 (DDIT3), a class of protein that play a role in ER stress. Induced DDIT3 inhibits the antiviral response in BVDB-infected cells by promoting NF-κB-dependent OTUD1. The upregulated OTUD1 act as a trigger factor to enhance the deubiquitylation of smurf1 and its protein level, a class of E3 enzymes of MAVS, mediating the degradation of the MAVS/TRAF3/TRAF6 signalosome and ultimately effectively inhibiting interferon production, ultimately dampening IFN-I production and promoting BVDV replication (Wang et al., 2021).

In particular, SARS-CoV-2, which has caused the current pandemic of coronavirus disease 2019 (COVID-19; Dong et al., 2020; Li et al., 2020), is inseparable from the damage to the host immune system. Lower level of IFNs production was found in SARS-CoV-2 infected cells and animal models compared to other respiratory viruses, as well as in the serum of COVID-19 patients (Blanco-Melo et al., 2020; Chu et al., 2020). The viral proteins such as non-structural proteins (nsp6, nsp13, and nsp15) and the accessory protein ORF9B are able to destroy the role of innate immune signaling proteins and hinder the transmission of antiviral signals (Hackbart et al., 2020; Lei et al., 2020; Xia et al., 2020; Wu et al., 2021). The PLpro, which is a part of the viral nsp3, endows the DUB activity (Barretto et al., 2005; Klemm et al., 2020). PLpro predominantly targets the ubiquitin-like interferon-stimulated gene 15 protein (ISG15), unraveling ISG15 from MDA5 or IRF3 (Shin et al., 2020; Liu et al., 2021). PLpro can also directly cleave IRF3 and weaken the production of IFNs (Moustaqil et al., 2021).

In addition, SARS-CoV-2 infection is able to induce RIG-I degradation through increasing the expression of the USP5. USP5 interacts with STUB1 (the E3 ligase of RIG-I) to catalyze the K48-linked ubiquitination of RIG-I (Zhang et al., 2021a). The nsp13 has been found to interact with TBK1 and interrupt the association between TBK1 and TRAFs, which inhibits the recruitment of TBK1 by MAVS and the activation of downstream signaling. The immune inhibitory effect of nsp13 on interferons was supported by USP13, which is a SARS-CoV-2 interacting protein that enriched in T cells (Sut, 2020), through deubiquitinating and stabilizing the expression of nsp13. Indeed, loss of USP13 or treatment with USP13 inhibitor leads to more IFNs production and less viral replication (Guo et al., 2021a).

All the above studies have shown that using cellular DUBs to target the proteins in innate immune signaling is an efficient aspect for viruses, including SARS-CoV-2, to evade host immune surveillance and benefit for the viral survival.

## Conclusion and Perspective

Deubiquitylating enzymes control PRR signaling and NLRP3 inflammasomes activation to ensure effective signal transduction and virus clearance. In antiviral innate immunity, recognizing of diversity polyubiquitin chains is the main mechanism for DUBs, their regulation of innate immune responses is subtle, tightly controlling signal activation at every stage, though this regulation is cannot be completed independently by one DUB in some cases and some other factors including post-translational modification can also regulate the catalytic activity of DUBs, which further affects the regulation of DUBs on substrate molecules. Despite DUBs playing an important role in regulating immune signaling, our knowledge about the regulation of antiviral innate immunity of DUBs family is still limited, and their specific regulatory mechanisms of DUBs remain to be fully elucidated.

On the other hand, the regulation of DUBs on the immune signal serves the host and can be used by the virus for its latent and replication. It is of great significance to develop specific drugs for anti-virus therapy targeting these DUB used by the virus.

## Author Contributions

SL conceived and wrote the manuscript. JL revised and designed this review. LY and WD revised the manuscript. All authors contributed to the article and approved the submitted version.

## Funding

This study was supported by the National Key Research and Development Program of China (2017YFC1200204), Natural Science Foundations of China (81974427), and Graduate Research and Innovation Projects of Central South University (2021zzts0931).

## Conflict of Interest

The authors declare that the research was conducted in the absence of any commercial or financial relationships that could be construed as a potential conflict of interest.

## Publisher’s Note

All claims expressed in this article are solely those of the authors and do not necessarily represent those of their affiliated organizations, or those of the publisher, the editors and the reviewers. Any product that may be evaluated in this article, or claim that may be made by its manufacturer, is not guaranteed or endorsed by the publisher.

## References

[ref1] Aeschlimann F. A. Batu E. D. Canna S. W. Go E. Gul A. Hoffmann P.. (2018). A20 haploinsufficiency (HA20): clinical phenotypes and disease course of patients with a newly recognised NF-kB-mediated autoinflammatory disease. Ann. Rheum. Dis. 77, 728–735. doi: 10.1136/annrheumdis-2017-212403, PMID: 29317407

[ref2] Akira S. Uematsu S. Takeuchi O. (2006). Pathogen recognition and innate immunity. Cell 124, 783–801. doi: 10.1016/j.cell.2006.02.015, PMID: 16497588

[ref3] Barretto N. Jukneliene D. Ratia K. Chen Z. Mesecar A. D. Baker S. C. (2005). The papain-like protease of severe acute respiratory syndrome coronavirus has deubiquitinating activity. J. Virol. 79, 15189–15198. doi: 10.1128/JVI.79.24.15189-15198.2005, PMID: 16306590 PMC1316023

[ref4] Bednash J. S. Johns F. Patel N. Smail T. R. Londino J. D. Mallampalli R. K. (2021). The deubiquitinase STAMBP modulates cytokine secretion through the NLRP3 inflammasome. Cell Signal. 79:109859. doi: 10.1016/j.cellsig.2020.109859, PMID: 33253913 PMC10201604

[ref5] Bignell G. R. Warren W. Seal S. Takahashi M. Rapley E. Barfoot R.. (2000). Identification of the familial cylindromatosis tumour-suppressor gene. Nat. Genet. 25, 160–165. doi: 10.1038/76006, PMID: 10835629

[ref6] Bilguvar K. Tyagi N. K. Ozkara C. Tuysuz B. Bakircioglu M. Choi M.. (2013). Recessive loss of function of the neuronal ubiquitin hydrolase UCHL1 leads to early-onset progressive neurodegeneration. Proc. Natl. Acad. Sci. U. S. A. 110, 3489–3494. doi: 10.1073/pnas.1222732110, PMID: 23359680 PMC3587195

[ref7] Blanco-Melo D. Nilsson-Payant B. E. Liu W. C. Uhl S. Hoagland D. Moller R.. (2020). Imbalanced host response to SARS-CoV-2 drives development of COVID-19. Cell 181, 1036.e9–1045.e9. doi: 10.1016/j.cell.2020.04.026, PMID: 32416070 PMC7227586

[ref8] Boone D. L. Turer E. E. Lee E. G. Ahmad R. C. Wheeler M. T. Tsui C.. (2004). The ubiquitin-modifying enzyme A20 is required for termination of toll-like receptor responses. Nat. Immunol. 5, 1052–1060. doi: 10.1038/ni1110, PMID: 15334086

[ref9] Broz P. Dixit V. M. (2016). Inflammasomes: mechanism of assembly, regulation and signalling. Nat. Rev. Immunol. 16, 407–420. doi: 10.1038/nri.2016.58, PMID: 27291964

[ref10] Cai J. Chen H. Y. Peng S. J. Meng J. L. Wang Y. Zhou Y.. (2018). USP7-TRIM27 axis negatively modulates antiviral type I IFN signaling. FASEB J. 32, 5238–5249. doi: 10.1096/fj.201700473RR, PMID: 29688809

[ref11] Cai Z. Zhang M. X. Tang Z. Zhang Q. Ye J. Xiong T. C.. (2020). USP22 promotes IRF3 nuclear translocation and antiviral responses by deubiquitinating the importin protein KPNA2. J. Exp. Med. 217:e20191174. doi: 10.1084/jem.20191174, PMID: 32130408 PMC7201923

[ref12] Cai B. Zhao J. Zhang Y. Liu Y. Ma C. Yi F.. (2021). USP5 attenuates NLRP3 inflammasome activation by promoting autophagic degradation of NLRP3. Autophagy 5, 1–15. doi: 10.1080/15548627.2021.1965426, PMID: 34486483 PMC9196652

[ref13] Chen Z. J. (2012). Ubiquitination in signaling to and activation of IKK. Immunol. Rev. 246, 95–106. doi: 10.1111/j.1600-065X.2012.01108.x, PMID: 22435549 PMC3549672

[ref14] Chen M. Meng Q. Qin Y. Liang P. Tan P. He L.. (2016). TRIM14 inhibits cGAS degradation mediated by selective autophagy receptor p62 to promote innate immune responses. Mol. Cell 64, 105–119. doi: 10.1016/j.molcel.2016.08.025, PMID: 27666593

[ref15] Chen X. Yang X. Zheng Y. Yang Y. Xing Y. Chen Z. (2014). SARS coronavirus papain-like protease inhibits the type I interferon signaling pathway through interaction with the STING-TRAF3-TBK1 complex. Protein Cell 5, 369–381. doi: 10.1007/s13238-014-0026-3, PMID: 24622840 PMC3996160

[ref16] Chiang C. Pauli E. K. Biryukov J. Feister K. F. Meng M. White E. A.. (2018). The human papillomavirus E6 oncoprotein targets USP15 and TRIM25 to suppress RIG-I-mediated innate immune signaling. J. Virol. 92, e01737–e01817. doi: 10.1128/JVI.01737-17, PMID: 29263274 PMC5827370

[ref17] Chu H. Chan J. F. Wang Y. Yuen T. T. Chai Y. Hou Y.. (2020). Comparative replication and immune activation profiles of SARS-CoV-2 and SARS-CoV in human lungs: an ex vivo study with implications for the pathogenesis of COVID-19. Clin. Infect. Dis. 71, 1400–1409. doi: 10.1093/cid/ciaa410, PMID: 32270184 PMC7184390

[ref18] Clague M. J. Urbe S. Komander D. (2019). Breaking the chains: deubiquitylating enzyme specificity begets function. Nat. Rev. Mol. Cell Biol. 20, 338–352. doi: 10.1038/s41580-019-0099-1, PMID: 30733604

[ref19] Coornaert B. Carpentier I. Beyaert R. (2009). A20: central gatekeeper in inflammation and immunity. J. Biol. Chem. 284, 8217–8221. doi: 10.1074/jbc.R800032200, PMID: 19008218 PMC2659177

[ref20] Dang W. Cao P. Yan Q. Yang L. Wang Y. Yang J.. (2021). IGFBP7-AS1 is a p53-responsive long noncoding RNA downregulated by epstein-barr virus that contributes to viral tumorigenesis. Cancer Lett. 523, 135–147. doi: 10.1016/j.canlet.2021.10.006, PMID: 34634383

[ref21] Daubeuf S. Singh D. Tan Y. Liu H. Federoff H. J. Bowers W. J.. (2009). HSV ICP0 recruits USP7 to modulate TLR-mediated innate response. Blood 113, 3264–3275. doi: 10.1182/blood-2008-07-168203, PMID: 18952891 PMC3401030

[ref22] Dong E. Du H. Gardner L. (2020). An interactive web-based dashboard to track COVID-19 in real time. Lancet Infect. Dis. 20, 533–534. doi: 10.1016/S1473-3099(20)30120-1, PMID: 32087114 PMC7159018

[ref23] Duong B. H. Onizawa M. Oses-Prieto J. A. Advincula R. Burlingame A. Malynn B. A.. (2015). A20 restricts ubiquitination of pro-interleukin-1beta protein complexes and suppresses NLRP3 inflammasome activity. Immunity 42, 55–67. doi: 10.1016/j.immuni.2014.12.031, PMID: 25607459 PMC4302274

[ref24] Elliott P. R. Leske D. Wagstaff J. Schlicher L. Berridge G. Maslen S.. (2021). Regulation of CYLD activity and specificity by phosphorylation and ubiquitin-binding CAP-Gly domains. Cell Rep. 37:109777. doi: 10.1016/j.celrep.2021.109777, PMID: 34610306 PMC8511506

[ref25] Fang R. Jiang Q. Zhou X. Wang C. Guan Y. Tao J.. (2017). MAVS activates TBK1 and IKKepsilon through TRAFs in NEMO dependent and independent manner. PLoS Pathog. 13:e1006720. doi: 10.1371/journal.ppat.1006720, PMID: 29125880 PMC5699845

[ref26] Feng W. Sun X. Shi N. Zhang M. Guan Z. Duan M. (2017). Influenza a virus NS1 protein induced A20 contributes to viral replication by suppressing interferon-induced antiviral response. Biochem. Biophys. Res. Commun. 482, 1107–1113. doi: 10.1016/j.bbrc.2016.11.166, PMID: 27914808

[ref27] Friedman C. S. O’donnell M. A. Legarda-Addison D. Ng A. Cardenas W. B. Yount J. S.. (2008). The tumour suppressor CYLD is a negative regulator of RIG-I-mediated antiviral response. EMBO Rep. 9, 930–936. doi: 10.1038/embor.2008.136, PMID: 18636086 PMC2529351

[ref28] Gack M. U. Shin Y. C. Joo C. H. Urano T. Liang C. Sun L.. (2007). TRIM25 RING-finger E3 ubiquitin ligase is essential for RIG-I-mediated antiviral activity. Nature 446, 916–920. doi: 10.1038/nature05732, PMID: 17392790

[ref29] Genin P. Cuvelier F. Lambin S. Corte-Real Filipe J. Autrusseau E. Laurent C.. (2015). Optineurin regulates the interferon response in a cell cycle-dependent manner. PLoS Pathog. 11:e1004877. doi: 10.1371/journal.ppat.1004971, PMID: 25923723 PMC4414543

[ref30] Guo G. Gao M. Gao X. Zhu B. Huang J. Luo K.. (2021a). SARS-CoV-2 non-structural protein 13 (nsp13) hijacks host deubiquitinase USP13 and counteracts host antiviral immune response. Signal Transduct. Target. Ther. 6:119. doi: 10.1038/s41392-021-00509-3, PMID: 33707416 PMC7947159

[ref31] Guo Y. Jiang F. Kong L. Li B. Yang Y. Zhang L.. (2019). Cutting edge: USP27X deubiquitinates and stabilizes the DNA sensor cGAS to regulate cytosolic DNA-mediated signaling. J. Immunol. 203, 2049–2054. doi: 10.4049/jimmunol.1900514, PMID: 31534008

[ref32] Guo Y. Jiang F. Kong L. Wu H. Zhang H. Chen X.. (2021b). OTUD5 promotes innate antiviral and antitumor immunity through deubiquitinating and stabilizing STING. Cell. Mol. Immunol. 18, 1945–1955. doi: 10.1038/s41423-020-00531-5, PMID: 32879469 PMC8322343

[ref33] Hackbart M. Deng X. Baker S. C. (2020). Coronavirus endoribonuclease targets viral polyuridine sequences to evade activating host sensors. Proc. Natl. Acad. Sci. U. S. A. 117, 8094–8103. doi: 10.1073/pnas.1921485117, PMID: 32198201 PMC7149396

[ref34] Hershko A. Ciechanover A. (1998). The ubiquitin system. Annu. Rev. Biochem. 67, 425–479. doi: 10.1146/annurev.biochem.67.1.425, PMID: 9759494

[ref35] Hou J. Han L. Zhao Z. Liu H. Zhang L. Ma C.. (2021). USP18 positively regulates innate antiviral immunity by promoting K63-linked polyubiquitination of MAVS. Nat. Commun. 12:2970. doi: 10.1038/s41467-021-23219-4, PMID: 34016972 PMC8137702

[ref36] Huang L. Liu H. Zhang K. Meng Q. Hu L. Zhang Y.. (2020). Ubiquitin-conjugating enzyme 2S enhances viral replication by inhibiting type I IFN production through recruiting USP15 to deubiquitinate TBK1. Cell Rep. 32:108044. doi: 10.1016/j.celrep.2020.108044, PMID: 32814047

[ref37] Hutti J. E. Shen R. R. Abbott D. W. Zhou A. Y. Sprott K. M. Asara J. M.. (2009). Phosphorylation of the tumor suppressor CYLD by the breast cancer oncogene IKKepsilon promotes cell transformation. Mol. Cell 34, 461–472. doi: 10.1016/j.molcel.2009.04.031, PMID: 19481526 PMC2746958

[ref38] Hutti J. E. Turk B. E. Asara J. M. Ma A. Cantley L. C. Abbott D. W. (2007). IkappaB kinase beta phosphorylates the K63 deubiquitinase A20 to cause feedback inhibition of the NF-kappaB pathway. Mol. Cell. Biol. 27, 7451–7461. doi: 10.1128/MCB.01101-07, PMID: 17709380 PMC2169042

[ref39] Inn K. S. Gack M. U. Tokunaga F. Shi M. Wong L. Y. Iwai K.. (2011). Linear ubiquitin assembly complex negatively regulates RIG-I- and TRIM25-mediated type I interferon induction. Mol. Cell 41, 354–365. doi: 10.1016/j.molcel.2010.12.029, PMID: 21292167 PMC3070481

[ref40] Jahan A. S. Biquand E. Munoz-Moreno R. Le Quang A. Mok C. K. Wong H. H.. (2020). OTUB1 is a key regulator of RIG-I-dependent immune signaling and is targeted for proteasomal degradation by influenza A NS1. Cell Rep. 30, 1570.e6–1584.e6. doi: 10.1016/j.celrep.2020.01.015, PMID: 32023470

[ref41] Jin J. Hu H. Li H. S. Yu J. Xiao Y. Brittain G. C.. (2014). Noncanonical NF-kappaB pathway controls the production of type I interferons in antiviral innate immunity. Immunity 40, 342–354. doi: 10.1016/j.immuni.2014.02.006, PMID: 24656046 PMC3983709

[ref42] Karim R. Tummers B. Meyers C. Biryukov J. L. Alam S. Backendorf C.. (2013). Human papillomavirus (HPV) upregulates the cellular deubiquitinase UCHL1 to suppress the keratinocyte’s innate immune response. PLoS Pathog. 9:e1003384. doi: 10.1371/journal.ppat.1003384, PMID: 23717208 PMC3662672

[ref43] Kayagaki N. Phung Q. Chan S. Chaudhari R. Quan C. O’rourke K. M.. (2007). DUBA: a deubiquitinase that regulates type I interferon production. Science 318, 1628–1632. doi: 10.1126/science.1145918, PMID: 17991829

[ref44] Klemm T. Ebert G. Calleja D. J. Allison C. C. Richardson L. W. Bernardini J. P.. (2020). Mechanism and inhibition of the papain-like protease, PLpro, of SARS-CoV-2. EMBO J. 39:e106275. doi: 10.15252/embj.2020106275, PMID: 32845033 PMC7461020

[ref45] Komander D. Clague M. J. Urbe S. (2009). Breaking the chains: structure and function of the deubiquitinases. Nat. Rev. Mol. Cell Biol. 10, 550–563. doi: 10.1038/nrm2731, PMID: 19626045

[ref46] Komander D. Lord C. J. Scheel H. Swift S. Hofmann K. Ashworth A.. (2008). The structure of the CYLD USP domain explains its specificity for Lys63-linked polyubiquitin and reveals a B box module. Mol. Cell 29, 451–464. doi: 10.1016/j.molcel.2007.12.018, PMID: 18313383

[ref47] Kovalenko A. Chable-Bessia C. Cantarella G. Israel A. Wallach D. Courtois G. (2003). The tumour suppressor CYLD negatively regulates NF-kappaB signalling by deubiquitination. Nature 424, 801–805. doi: 10.1038/nature01802, PMID: 12917691

[ref48] Krug R. M. (2015). Functions of the influenza A virus NS1 protein in antiviral defense. Curr. Opin. Virol. 12, 1–6. doi: 10.1016/j.coviro.2015.01.007, PMID: 25638592 PMC4470714

[ref49] Kumari P. Kumar H. (2018). Viral deubiquitinases: role in evasion of anti-viral innate immunity. Crit. Rev. Microbiol. 44, 304–317. doi: 10.1080/1040841X.2017.1368999, PMID: 28885059

[ref50] Laherty C. D. Hu H. M. Opipari A. W. Wang F. Dixit V. M. (1992). The epstein-barr virus LMP1 gene product induces A20 zinc finger protein expression by activating nuclear factor kappa B. J. Biol. Chem. 267, 24157–24160. doi: 10.1016/S0021-9258(18)35741-7, PMID: 1332946

[ref51] Lee J. Y. Seo D. You J. Chung S. Park J. S. Lee J. H.. (2017). The deubiquitinating enzyme, ubiquitin-specific peptidase 50, regulates inflammasome activation by targeting the ASC adaptor protein. FEBS Lett. 591, 479–490. doi: 10.1002/1873-3468.12558, PMID: 28094437 PMC5324553

[ref52] Lei X. Dong X. Ma R. Wang W. Xiao X. Tian Z.. (2020). Activation and evasion of type I interferon responses by SARS-CoV-2. Nat. Commun. 11:3810. doi: 10.1038/s41467-020-17665-9, PMID: 32733001 PMC7392898

[ref53] Leroy E. Boyer R. Auburger G. Leube B. Ulm G. Mezey E.. (1998). The ubiquitin pathway in Parkinson’s disease. Nature 395, 451–452. doi: 10.1038/26652, PMID: 9774100

[ref54] Li S. Wang L. Berman M. Kong Y. Y. Dorf M. E. (2011). Mapping a dynamic innate immunity protein interaction network regulating type I interferon production. Immunity 35, 426–440. doi: 10.1016/j.immuni.2011.06.014, PMID: 21903422 PMC3253658

[ref55] Li Y. Zhang Z. Yang L. Lian X. Xie Y. Li S.. (2020). The MERS-CoV receptor DPP4 as a candidate binding target of the SARS-CoV-2 spike. iScience 23:101160. doi: 10.1016/j.isci.2020.101160, PMID: 32405622 PMC7219414

[ref56] Li H. Zhao Z. Ling J. Pan L. Zhao X. Zhu H.. (2019a). USP14 promotes K63-linked RIG-I deubiquitination and suppresses antiviral immune responses. Eur. J. Immunol. 49, 42–53. doi: 10.1002/eji.201847603, PMID: 30466171

[ref57] Li S. Zheng J. Chai L. Lin M. Zeng R. Lu J.. (2019b). Rapid and efficient differentiation of rodent neural stem cells into oligodendrocyte progenitor cells. Dev. Neurosci. 41, 79–93. doi: 10.1159/000499364, PMID: 31167194

[ref58] Li S. Zheng H. Mao A. P. Zhong B. Li Y. Liu Y.. (2010). Regulation of virus-triggered signaling by OTUB1- and OTUB2-mediated deubiquitination of TRAF3 and TRAF6. J. Biol. Chem. 285, 4291–4297. doi: 10.1074/jbc.M109.074971, PMID: 19996094 PMC2836033

[ref59] Lin S. Yang S. He J. Guest J. D. Ma Z. Yang L.. (2019). Zika virus NS5 protein antagonizes type I interferon production via blocking TBK1 activation. Virology 527, 180–187. doi: 10.1016/j.virol.2018.11.009, PMID: 30530224 PMC6340140

[ref60] Lin W. Zhang J. Lin H. Li Z. Sun X. Xin D.. (2016b). Syndecan-4 negatively regulates antiviral signalling by mediating RIG-I deubiquitination via CYLD. Nat. Commun. 7:11848. doi: 10.1038/ncomms11848, PMID: 27279133 PMC4906230

[ref61] Lin M. Zhao Z. Yang Z. Meng Q. Tan P. Xie W.. (2016a). USP38 inhibits type I interferon signaling by editing TBK1 ubiquitination through NLRP4 signalosome. Mol. Cell 64, 267–281. doi: 10.1016/j.molcel.2016.08.029, PMID: 27692986

[ref62] Liu H. Fan J. Zhang W. Chen Q. Zhang Y. Wu Z. (2020). OTUD4 alleviates hepatic ischemia-reperfusion injury by suppressing the K63-linked ubiquitination of TRAF6. Biochem. Biophys. Res. Commun. 523, 924–930. doi: 10.1016/j.bbrc.2019.12.114, PMID: 31964525

[ref63] Liu G. Lee J. H. Parker Z. M. Acharya D. Chiang J. J. Van Gent M.. (2021). ISG15-dependent activation of the sensor MDA5 is antagonized by the SARS-CoV-2 papain-like protease to evade host innate immunity. Nat. Microbiol. 6, 467–478. doi: 10.1038/s41564-021-00884-1, PMID: 33727702 PMC8103894

[ref64] Liu X. Li H. Zhong B. Blonska M. Gorjestani S. Yan M.. (2013). USP18 inhibits NF-kappaB and NFAT activation during Th17 differentiation by deubiquitinating the TAK1-TAB1 complex. J. Exp. Med. 210, 1575–1590. doi: 10.1084/jem.20122327, PMID: 23825189 PMC3727316

[ref65] Liu Y. C. Penninger J. Karin M. (2005). Immunity by ubiquitylation: a reversible process of modification. Nat. Rev. Immunol. 5, 941–952. doi: 10.1038/nri1731, PMID: 16322747 PMC7096784

[ref66] Liuyu T. Yu K. Ye L. Zhang Z. Zhang M. Ren Y.. (2019). Induction of OTUD4 by viral infection promotes antiviral responses through deubiquitinating and stabilizing MAVS. Cell Res. 29, 67–79. doi: 10.1038/s41422-018-0107-6, PMID: 30410068 PMC6318273

[ref123] Lork M. Kreike M. Staal J. Beyaert R. (2018). Importance of validating antibodies and small compound inhibitors uing genetic knockout studies-T cell receptor-induced CYLD phosphorylation by IKKepsilon/TBK1 as a case study. Front. Cell Dev. Biol. 6:40. doi: 10.3389/fcell.2018.00040 29755980 PMC5932415

[ref67] Maelfait J. Roose K. Bogaert P. Sze M. Saelens X. Pasparakis M.. (2012). A20 (Tnfaip3) deficiency in myeloid cells protects against influenza A virus infection. PLoS Pathog. 8:e1002570. doi: 10.1371/journal.ppat.1002570, PMID: 22396652 PMC3291650

[ref68] Malakhova O. A. Kim K. I. Luo J. K. Zou W. Kumar K. G. Fuchs S. Y.. (2006). UBP43 is a novel regulator of interferon signaling independent of its ISG15 isopeptidase activity. EMBO J. 25, 2358–2367. doi: 10.1038/sj.emboj.7601149, PMID: 16710296 PMC1478183

[ref69] Man S. M. Kanneganti T. D. (2016). Converging roles of caspases in inflammasome activation, cell death and innate immunity. Nat. Rev. Immunol. 16, 7–21. doi: 10.1038/nri.2015.7, PMID: 26655628 PMC4915362

[ref70] Mevissen T. E. T. Komander D. (2017). Mechanisms of deubiquitinase specificity and regulation. Annu. Rev. Biochem. 86, 159–192. doi: 10.1146/annurev-biochem-061516-044916, PMID: 28498721

[ref71] Moustaqil M. Ollivier E. Chiu H. P. Van Tol S. Rudolffi-Soto P. Stevens C.. (2021). SARS-CoV-2 proteases PLpro and 3CLpro cleave IRF3 and critical modulators of inflammatory pathways (NLRP12 and TAB1): implications for disease presentation across species. Emerg. Microbes Infect. 10, 178–195. doi: 10.1080/22221751.2020.1870414, PMID: 33372854 PMC7850364

[ref72] Ndoja A. Cohen R. E. Yao T. (2014). Ubiquitin signals proteolysis-independent stripping of transcription factors. Mol. Cell 53, 893–903. doi: 10.1016/j.molcel.2014.02.002, PMID: 24613342 PMC4005849

[ref73] Ning S. Huye L. E. Pagano J. S. (2005). Interferon regulatory factor 5 represses expression of the epstein-barr virus oncoprotein LMP1: braking of the IRF7/LMP1 regulatory circuit. J. Virol. 79, 11671–11676. doi: 10.1128/JVI.79.18.11671-11676.2005, PMID: 16140744 PMC1212628

[ref74] Ning S. Pagano J. S. (2010). The A20 deubiquitinase activity negatively regulates LMP1 activation of IRF7. J. Virol. 84, 6130–6138. doi: 10.1128/JVI.00364-10, PMID: 20392859 PMC2876664

[ref75] Ohtake F. Saeki Y. Ishido S. Kanno J. Tanaka K. (2016). The K48-K63 branched ubiquitin chain regulates NF-kappaB signaling. Mol. Cell 64, 251–266. doi: 10.1016/j.molcel.2016.09.014, PMID: 27746020

[ref76] Palazon-Riquelme P. Worboys J. D. Green J. Valera A. Martin-Sanchez F. Pellegrini C.. (2018). USP7 and USP47 deubiquitinases regulate NLRP3 inflammasome activation. EMBO Rep. 19:e44766. doi: 10.15252/embr.201744766, PMID: 30206189 PMC6172458

[ref77] Parvatiyar K. Barber G. N. Harhaj E. W. (2010). TAX1BP1 and A20 inhibit antiviral signaling by targeting TBK1-IKKi kinases. J. Biol. Chem. 285, 14999–15009. doi: 10.1074/jbc.M110.109819, PMID: 20304918 PMC2865285

[ref78] Pauli E. K. Chan Y. K. Davis M. E. Gableske S. Wang M. K. Feister K. F.. (2014). The ubiquitin-specific protease USP15 promotes RIG-I-mediated antiviral signaling by deubiquitylating TRIM25. Sci. Signal. 7:ra3. doi: 10.1126/scisignal.2004577, PMID: 24399297 PMC4008495

[ref79] Py B. F. Kim M. S. Vakifahmetoglu-Norberg H. Yuan J. (2013). Deubiquitination of NLRP3 by BRCC3 critically regulates inflammasome activity. Mol. Cell 49, 331–338. doi: 10.1016/j.molcel.2012.11.009, PMID: 23246432

[ref80] Rajamaki K. Keskitalo S. Seppanen M. Kuismin O. Vahasalo P. Trotta L.. (2018). Haploinsufficiency of A20 impairs protein-protein interactome and leads into caspase-8-dependent enhancement of NLRP3 inflammasome activation. RMD Open 4:e000740. doi: 10.1136/rmdopen-2018-000740, PMID: 30402268 PMC6203104

[ref81] Reiley W. Zhang M. Y. Wu X. F. Granger E. Sun S. C. (2005). Regulation of the deubiquitinating enzyme CYLD by I kappa B kinase gamma-dependent phosphorylation. Mol. Cell. Biol. 25, 3886–3895. doi: 10.1128/MCB.25.10.3886-3895.2005, PMID: 15870263 PMC1087725

[ref82] Ren G. Zhang X. Xiao Y. Zhang W. Wang Y. Ma W.. (2019). ABRO1 promotes NLRP3 inflammasome activation through regulation of NLRP3 deubiquitination. EMBO J. 38:e100376. doi: 10.15252/embj.2018100376, PMID: 30787184 PMC6418445

[ref83] Sato Y. Goto E. Shibata Y. Kubota Y. Yamagata A. Goto-Ito S.. (2015). Structures of CYLD USP with Met1- or Lys63-linked diubiquitin reveal mechanisms for dual specificity. Nat. Struct. Mol. Biol. 22, 222–229. doi: 10.1038/nsmb.2970, PMID: 25686088

[ref84] Shin D. Mukherjee R. Grewe D. Bojkova D. Baek K. Bhattacharya A.. (2020). Papain-like protease regulates SARS-CoV-2 viral spread and innate immunity. Nature 587, 657–662. doi: 10.1038/s41586-020-2601-5, PMID: 32726803 PMC7116779

[ref85] Siu K. L. Yuen K. S. Castano-Rodriguez C. Ye Z. W. Yeung M. L. Fung S. Y.. (2019). Severe acute respiratory syndrome coronavirus ORF3a protein activates the NLRP3 inflammasome by promoting TRAF3-dependent ubiquitination of ASC. FASEB J. 33, 8865–8877. doi: 10.1096/fj.201802418R, PMID: 31034780 PMC6662968

[ref86] Song H. Zhao C. Yu Z. Li Q. Yan R. Qin Y.. (2020). UAF1 deubiquitinase complexes facilitate NLRP3 inflammasome activation by promoting NLRP3 expression. Nat. Commun. 11:6042. doi: 10.1038/s41467-020-19939-8, PMID: 33247121 PMC7695691

[ref87] Sui L. Zhao Y. Wang W. Wu P. Wang Z. Yu Y.. (2021). SARS-CoV-2 membrane protein inhibits type I interferon production through ubiquitin-mediated degradation of TBK1. Front. Immunol. 12:662989. doi: 10.3389/fimmu.2021.662989, PMID: 34084167 PMC8168463

[ref88] Sun H. Zhang Q. Jing Y. Y. Zhang M. Wang H. Y. Cai Z.. (2017). USP13 negatively regulates antiviral responses by deubiquitinating STING. Nat. Commun. 8:15534. doi: 10.1038/ncomms15534, PMID: 28534493 PMC5457515

[ref89] Sut B. B. (2020). Molecular profiling of immune cell-enriched severe acute respiratory syndrome coronavirus 2 (SARS-CoV-2) interacting protein USP13. Life Sci. 258:118170. doi: 10.1016/j.lfs.2020.118170, PMID: 32735883 PMC7387267

[ref90] Takeuchi O. Akira S. (2010). Pattern recognition receptors and inflammation. Cell 140, 805–820. doi: 10.1016/j.cell.2010.01.022, PMID: 20303872

[ref91] Tao X. Chu B. Xin D. Li L. Sun Q. (2020). USP27X negatively regulates antiviral signaling by deubiquitinating RIG-I. PLoS Pathog. 16:e1008293. doi: 10.1371/journal.ppat.1008293, PMID: 32027733 PMC7029883

[ref92] Tate M. D. Mansell A. (2018). An update on the NLRP3 inflammasome and influenza: the road to redemption or perdition? Curr. Opin. Immunol. 54, 80–85. doi: 10.1016/j.coi.2018.06.005, PMID: 29986838

[ref93] Walle L. V. Van Opdenbosch N. Jacques P. Fossoul A. Verheugen E. Vogel P.. (2014). Negative regulation of the NLRP3 inflammasome by A20 protects against arthritis. Nature 512, 69–73. doi: 10.1038/nature13322, PMID: 25043000 PMC4126806

[ref94] Wang D. Fang L. Li P. Sun L. Fan J. Zhang Q.. (2011). The leader proteinase of foot-and-mouth disease virus negatively regulates the type I interferon pathway by acting as a viral deubiquitinase. J. Virol. 85, 3758–3766. doi: 10.1128/JVI.02589-10, PMID: 21307201 PMC3126127

[ref95] Wang S. Hou P. Pan W. He W. He D. C. Wang H.. (2021). DDIT3 targets innate immunity via the DDIT3-OTUD1-MAVS pathway to promote bovine viral diarrhea virus replication. J. Virol. 95, e02351–e02320. doi: 10.1128/JVI.02351-20, PMID: 33361422 PMC8094964

[ref96] Wang W. Li G. De W. Luo Z. Pan P. Tian M.. (2018). Zika virus infection induces host inflammatory responses by facilitating NLRP3 inflammasome assembly and interleukin-1beta secretion. Nat. Commun. 9:106. doi: 10.1038/s41467-017-02645-3, PMID: 29317641 PMC5760693

[ref97] Wu J. Shi Y. Pan X. Wu S. Hou R. Zhang Y.. (2021). SARS-CoV-2 ORF9b inhibits RIG-I-MAVS antiviral signaling by interrupting K63-linked ubiquitination of NEMO. Cell Rep. 34:108761. doi: 10.1016/j.celrep.2021.108761, PMID: 33567255 PMC7857071

[ref98] Xia H. Cao Z. Xie X. Zhang X. Chen J. Y. Wang H.. (2020). Evasion of type I interferon by SARS-CoV-2. Cell Rep. 33:108234. doi: 10.1016/j.celrep.2020.108234, PMID: 32979938 PMC7501843

[ref99] Xin S. Du S. Liu L. Xie Y. Zuo L. Yang J.. (2019). Epstein-barr virus nuclear antigen 1 recruits cyclophilin A to facilitate the replication of viral DNA genome. Front. Microbiol. 10:2879. doi: 10.3389/fmicb.2019.02879, PMID: 31921057 PMC6923202

[ref100] Xue Q. Liu H. Zhu Z. Yang F. Xue Q. Cai X.. (2018). Seneca valley virus 3C protease negatively regulates the type I interferon pathway by acting as a viral deubiquitinase. Antivir. Res. 160, 183–189. doi: 10.1016/j.antiviral.2018.10.028, PMID: 30408499 PMC7111287

[ref101] Yang X. D. Li W. Zhang S. Wu D. Jiang X. Tan R.. (2020). PLK4 deubiquitination by Spata2-CYLD suppresses NEK7-mediated NLRP3 inflammasome activation at the centrosome. EMBO J. 39:e102201. doi: 10.15252/embj.2019102201, PMID: 31762063 PMC6960439

[ref102] Yang Z. Xian H. Hu J. Tian S. Qin Y. Wang R. F.. (2015). USP18 negatively regulates NF-kappaB signaling by targeting TAK1 and NEMO for deubiquitination through distinct mechanisms. Sci. Rep. 5:12738. doi: 10.1038/srep12738, PMID: 26240016 PMC4523862

[ref103] Yeh H. M. Yu C. Y. Yang H. C. Ko S. H. Liao C. L. Lin Y. L. (2013). Ubiquitin-specific protease 13 regulates IFN signaling by stabilizing STAT1. J. Immunol. 191, 3328–3336. doi: 10.4049/jimmunol.1300225, PMID: 23940278

[ref104] Yoshida H. Jono H. Kai H. Li J. D. (2005). The tumor suppressor cylindromatosis (CYLD) acts as a negative regulator for toll-like receptor 2 signaling via negative cross-talk with TRAF6 AND TRAF7. J. Biol. Chem. 280, 41111–41121. doi: 10.1074/jbc.M509526200, PMID: 16230348

[ref105] Yu Z. Song H. Jia M. Zhang J. Wang W. Li Q.. (2017). USP1-UAF1 deubiquitinase complex stabilizes TBK1 and enhances antiviral responses. J. Exp. Med. 214, 3553–3563. doi: 10.1084/jem.20170180, PMID: 29138248 PMC5716033

[ref106] Zeng W. Xu M. Liu S. Sun L. Chen Z. J. (2009). Key role of Ubc5 and lysine-63 polyubiquitination in viral activation of IRF3. Mol. Cell 36, 315–325. doi: 10.1016/j.molcel.2009.09.037, PMID: 19854139 PMC2779157

[ref107] Zhang L. Huang F. Liu J. Xu Y. Miao Y. Yuan Y.. (2021b). HSV-1-encoded ICP0 degrades the host deubiquitinase BRCC36 to antagonize interferon antiviral response. Mol. Immunol. 135, 28–35. doi: 10.1016/j.molimm.2021.03.027, PMID: 33857816

[ref108] Zhang J. Stirling B. Temmerman S. T. Ma C. A. Fuss I. J. Derry J. M.. (2006). Impaired regulation of NF-kappaB and increased susceptibility to colitis-associated tumorigenesis in CYLD-deficient mice. J. Clin. Invest. 116, 3042–3049. doi: 10.1172/JCI28746, PMID: 17053834 PMC1616194

[ref109] Zhang Z. Wang D. Wang P. Zhao Y. You F. (2020). OTUD1 negatively regulates type I IFN induction by disrupting noncanonical ubiquitination of IRF3. J. Immunol. 204, 1904–1918. doi: 10.4049/jimmunol.1900305, PMID: 32075857

[ref110] Zhang M. Wang L. Zhao X. Zhao K. Meng H. Zhao W.. (2012). TRAF-interacting protein (TRIP) negatively regulates IFN-beta production and antiviral response by promoting proteasomal degradation of TANK-binding kinase 1. J. Exp. Med. 209, 1703–1711. doi: 10.1084/jem.20120024, PMID: 22945920 PMC3457734

[ref111] Zhang H. Wang D. Zhong H. Luo R. Shang M. Liu D.. (2015). Ubiquitin-specific protease 15 negatively regulates virus-induced type I interferon signaling via catalytically-dependent and -independent mechanisms. Sci. Rep. 5:11220. doi: 10.1038/srep11220, PMID: 26061460 PMC4650652

[ref112] Zhang L. Wei N. Cui Y. Hong Z. Liu X. Wang Q.. (2018). The deubiquitinase CYLD is a specific checkpoint of the STING antiviral signaling pathway. PLoS Pathog. 14:e1007435. doi: 10.1371/journal.ppat.1007435, PMID: 30388174 PMC6235404

[ref113] Zhang M. Wu X. Lee A. J. Jin W. Chang M. Wright A.. (2008). Regulation of IkappaB kinase-related kinases and antiviral responses by tumor suppressor CYLD. J. Biol. Chem. 283, 18621–18626. doi: 10.1074/jbc.M801451200, PMID: 18467330 PMC2441564

[ref114] Zhang M. Zhang M. X. Zhang Q. Zhu G. F. Yuan L. Zhang D. E.. (2016). USP18 recruits USP20 to promote innate antiviral response through deubiquitinating STING/MITA. Cell Res. 26, 1302–1319. doi: 10.1038/cr.2016.125, PMID: 27801882 PMC5143414

[ref115] Zhang L. Zhao X. Zhang M. Zhao W. Gao C. (2014). Ubiquitin-specific protease 2b negatively regulates IFN-beta production and antiviral activity by targeting TANK-binding kinase 1. J. Immunol. 193, 2230–2237. doi: 10.4049/jimmunol.1302634, PMID: 25070846

[ref116] Zhang H. Zheng H. Zhu J. Dong Q. Wang J. Fan H.. (2021a). Ubiquitin-modified proteome of SARS-CoV-2-infected host cells reveals insights into virus-host interaction and pathogenesis. J. Proteome Res. 20, 2224–2239. doi: 10.1021/acs.jproteome.0c00758, PMID: 33666082

[ref117] Zhao X. Di Q. Yu J. Quan J. Xiao Y. Zhu H.. (2021). USP19 (ubiquitin specific peptidase 19) promotes TBK1 (TANK-binding kinase 1) degradation via chaperone-mediated autophagy. Autophagy 1–18. doi: 10.1080/15548627.2021.1963155, PMID: 34436957 PMC9037486

[ref118] Zhao Y. Mudge M. C. Soll J. M. Rodrigues R. B. Byrum A. K. Schwarzkopf E. A.. (2018). OTUD4 is a phospho-activated K63 deubiquitinase that regulates MyD88-dependent signaling. Mol. Cell 69:505.e5–516.e5. doi: 10.1016/j.molcel.2018.01.009, PMID: 29395066 PMC6819006

[ref119] Zheng Y. Liu Q. Wu Y. Ma L. Zhang Z. Liu T.. (2018). Zika virus elicits inflammation to evade antiviral response by cleaving cGAS via NS1-caspase-1 axis. EMBO J. 37:e99347. doi: 10.15252/embj.201899347, PMID: 30065070 PMC6138430

[ref120] Zhou Z. Cai X. Zhu J. Li Z. Yu G. Liu X.. (2021). Zebrafish otud6b negatively regulates antiviral responses by suppressing K63-linked ubiquitination of irf3 and irf7. J. Immunol. 207, 244–256. doi: 10.4049/jimmunol.2000891, PMID: 34183367

[ref121] Zhou Q. Cheng C. Wei Y. Yang J. Zhou W. Song Q.. (2020). USP15 potentiates NF-kappaB activation by differentially stabilizing TAB2 and TAB3. FEBS J. 287, 3165–3183. doi: 10.1111/febs.15202, PMID: 31903660

[ref122] Zong Z. Zhang Z. Wu L. Zhang L. Zhou F. (2021). The functional deubiquitinating enzymes in control of innate antiviral immunity. Adv. Sci. 8:2002484. doi: 10.1002/advs.202002484, PMID: 33511009 PMC7816709

